# Histologic chorioamnionitis does not modulate the oxidative stress and antioxidant status in pregnancies complicated by spontaneous preterm delivery

**DOI:** 10.1186/s12884-017-1549-4

**Published:** 2017-11-13

**Authors:** Laura Fernandes Martin, Natália Prearo Moço, Moisés Diôgo de Lima, Jossimara Polettini, Hélio Amante Miot, Camila Renata Corrêa, Ramkumar Menon, Márcia Guimarães da Silva

**Affiliations:** 1 Department of Pathology, Botucatu Medical School, São Paulo State University (UNESP), Distrito de Rubião Júnior, Botucatu, São Paulo CEP 18618-686 Brazil; 2 Department of Gynecology and Obstetrics, Federal University of Paraíba, UFPB, João Pessoa, Brazil; 3 The University of Western São Paulo, UNOESTE, Presidente Prudente, Brazil; 4 Department of Obstetrics & Gynecology, The University of Texas Medical Branch at Galveston, Galveston, TX USA

**Keywords:** Oxidative stress, Antioxidant capacity, Histologic chorioamnionitis, Preterm birth

## Abstract

**Background:**

Infection induced-inflammation and other risk factors for spontaneous preterm birth (PTB) and preterm premature rupture of membranes (pPROM) may cause a redox imbalance, increasing the release of free radicals and consuming antioxidant defenses. Oxidative stress, in turn, can initiate intracellular signaling cascades that increase the production of pro-inflammatory mediators.

The objective of this study was to evaluate the oxidative damage to proteins and antioxidant capacity profiles in amniochorion membranes from preterm birth (PTB) and preterm premature rupture of membranes (pPROM) and to determine the role of histologic chorioamnionitis in this scenario.

**Methods:**

We included 27 pregnant women with PTB, 27 pPROM and 30 at term. Protein oxidative damage was assayed by 3-nitrotyrosine (3-NT) and carbonyl levels, using enzyme-linked immunosorbent assay (ELISA) and modified dinitrophenylhydrazine assay (DNPH), respectively. Total antioxidant capacity (TAC) was measured by ELISA.

**Results:**

Protein oxidative damage determined by carbonyl levels was lower in PTB group than pPROM and term groups (*p* < 0.001). PTB group presented higher TAC compared with pPROM and term groups (*p* = 0.002). Histologic chorioamnionitis did not change either protein oxidative damage or TAC regardless of gestational outcome.

**Conclusion:**

These results corroborates previous reports that pPROM and term birth exhibit similarities in oxidative stress- induced senescence and histologic chorioamnionitis does not modulate oxidative stress or antioxidant status.

## Background

Reactive oxygen and nitrogen species (ROS and RNS), collectively named free radicals, are generated spontaneously in all aerobic organisms [1–4]. The mechanism termed redox balance ensures that the production of free radicals is not harmful to biological systems by preventing the excessive formation and action of ROS and RNS or by favoring the repair and reconstruction of biological structures that have been affected by them [5, 6]. Antioxidant defense mechanisms essential to redox balance are composed of enzymatic and non-enzymatic antioxidants. Enzymatic antioxidants include catalase, glutathione peroxidase (GPx), glutathione reductase (GSR) and superoxide dismutase (SOD). Non-enzymatic antioxidants include antioxidant compounds, such as glutathione and vitamins A, C and E [7]. Oxidative stress is an imbalance in redox status, towards excess ROS and RNS generation [6, 8, 9]. When the total antioxidant capacity (TAC) decreases or free radical levels increase [10], these exacerbated ROS and RNS formations can damage lipids, proteins and nucleic acids by modifying their expression, structure and function [11–13].

Oxidative stress damage to cells and tissues plays an important role in several pathological processes, including cardiovascular disease [14, 15], cancer [16–18], chronic inflammation [19], neurological disorders [20], metabolic syndrome [21, 22] and pregnancy complications [1, 6, 9, 23, 24]. Recent reports have linked spontaneous preterm birth (PTB) and preterm premature rupture of membranes (pPROM) pathophysiology to oxidative stress damage, where the latter is associated with oxidative stress-induced inflammation and considered as the disease of the fetal membranes [25, 26].

Despite the multifactorial etiology of PTB, intra-amniotic infection followed by maternal inflammatory response activation is reported as the major risk factor for PTB and is present in approximately 40% of preterm pregnancies [27–30]. pPROM in labor is associated with approximately 75% of infection [31]. The inflammatory response in the amniochorion membranes in response to bacterial infection can be diagnosed using clinical or histological criteria. Histologic chorioamnionitis (HC) is defined by the presence of polymorphonuclear cell (PMN) infiltrate in the amniochorion membranes [32].

Bacterial phagocytosis by PMNs during an inflammatory process results in an oxidative burst that is an antimicrobial mechanism characterized by the rapid generation and release of ROS and RNS, leading to oxidative stress [33]. Oxidative stress, in turn, can initiate intracellular signaling cascades that increase the production of pro-inflammatory mediators [34, 35]. Recent reports suggest that a substantial number of PTB and pPROM are associated with sterile inflammation in the absence of documented intra-amniotic infection or histologic chorioamnionitis. This is indicative of an alternate pathophysiology that can lead to an inflammatory process. Oxidative stress-induced senescence and senescence associated with inflammation, termed as sterile inflammation, has been linked to both PTB and pPROM. Both infectious and sterile inflammation exhibit a similar set of biomarkers. This study was conducted to assess oxidative stress-induced damage by determining protein peroxidation and total antioxidant capacity of fetal membranes from pregnancies complicated by PTB and pPROM compared with normal term pregnancies and evaluate the role of histologic chorioamnionitis in this scenario.

## Methods

The research project was approved by the Research Ethics Committee Board of the Federal University of Paraíba (UFPB), under protocol no. 1255858. Written informed consent was obtained from all the participants, and sociodemographic and behavioral data were acquired from patient medical records.

### Study population

This cross-sectional study was conducted in the Obstetrics Unit of the UFPB Lauro Wanderley Hospital, Paraíba State, Brazil, and in the Laboratory of Immunopathology of the Maternal-Fetal Relationship of the Department of Pathology of Botucatu Medical School, São Paulo State University (UNESP), São Paulo State, Brazil, from January to December 2014. Eighty-four pregnant women were enrolled. The preterm group was composed of 27 pregnant women who presented PTB with intact membranes and 27 pregnant women who presented pPROM. All these pregnant women delivered before 37 weeks of gestation. The term group was composed of 30 normal pregnant women in labor. Normal pregnancy was defined as singleton full-term and uneventful pregnancy, with no chronic or gestational medical conditions. The PTB, pPROM and term groups were subdivided according to the presence or absence of histologic chorioamnionitis.

The exclusion criteria were as follows: BMI > 30 kg/m^2^, preeclampsia, HELLP syndrome, gestational diabetes, hypertension, multiple pregnancies, cervical isthmus incompetence, placenta previa, placental abruption, RH-incompatibility, oligohydramnios or polyhydramnios, intrauterine growth restriction, malformation or fetal death, systemic infection, thyroid disease, HIV infection and drug and/or tobacco use.

Gestational age was calculated from the first day of the last menstrual period and/or by first-trimester ultrasound examination. Clinical diagnoses of PTB were made based on the following criteria: uterine contractions ≥4 per 20 min, cervical dilatation of at least 1 cm and cervical ripening [36]. pPROM was defined as the leakage of amniotic fluid prior to the onset of labor. This condition was confirmed with vaginal discharge pH ≥7 and/or positive result in an AmniSure test [37].

### Sample collection and histopathological analyses

Amniochorion membranes were collected and cleaned of blood clots and decidua in sterile conditions after placental delivery, flash frozen in liquid nitrogen and stored at −80 °C until processing. Samples of the membranes were fixed in 10% formalin, embedded in paraffin, sectioned and stained with hematoxylin and eosin for histopathological analyses. Histologic chorioamnionitis was diagnosed by the presence of neutrophilic infiltration in amniochorion membranes, as described by Redline et al. [32].

### Measurement of 3-nitrotyrosine levels

Protein peroxidation was determined using the 3-nitrotyrosine (3-NT) ELISA Kit, according to the manufacturer’s instructions (Abcam, Cambridge, UK). 3-NT is a product of protein tyrosine nitration resulting from oxidative damage to proteins by peroxynitrite, which can result in changes in protein structure, function and catalytic activity.

Amniochorion membrane sections of 25 mg were prepared for the assay by grinding with liquid nitrogen and homogenization with 1 mL of PBS. Next, aliquots were prepared containing 60 μL of tissue homogenate to which 240 μL of extraction buffer containing protease inhibitor were added. The samples were incubated on ice for 20 min. Tissue homogenates were centrifuged at 16000 x g for 10 min at 4 °C and the supernatants were collected immediately and stored at −20 °C.

The total protein concentration of all samples was measured by the Bradford protein assay, according to the manufacturer’s instructions (Bio-Rad, CA, USA), and then adjusted to 1 mg/mL.

A standard curve was obtained in parallel to each assay and the absorbance results were converted to pg/mL. At the end of the reaction, the absorbance was read spectrophotometrically at 450 nm in an automatic ELISA reader (Biotek Instruments Inc., Winooski, USA) and the concentration of 3-NT in each sample was determined by comparison with a standard curve. All the samples were tested in duplicate. The minimum detectable 3-NT level for assays was 0.053 ng/mL.

### Measurement of carbonyl levels

Protein carbonylation represents the most frequent and usually irreversible oxidative modification affecting proteins. In this study, a modified dinitrophenylhydrazine (DNPH) assay was performed. The principal method used to evaluate protein carbonylation is protein carbonyl derivatization with 2,4 DNPH, followed by spectrophotometric measurement, in which NaOH is added to the protein solution after the addition of DNPH, triggering an increase of the maximum absorbance wavelength of derivatized proteins from 370 to 450 nm. This increase minimizes the background of free DNPH that are read at 366–370 nm [38].

Amniochorion membrane sections of 25 mg were prepared for the assay by grinding with liquid nitrogen and homogenization with 1 mL of PBS containing the anti-protease cocktail. Tissue homogenates were centrifuged at 1600 x g for 10 min at 4 °C and the supernatants were immediately collected and stored at −20 °C. The total protein concentration of all samples was measured by the Pierce BCA assay, according to the manufacturer’s instructions (Thermo Fisher Scientific, MA, USA).

Aliquots of 100 μL were placed in a 96-well plate, and then 100 μL of DNPH solution (19.8 mg DNPH in 10 mL HCl2M) were added. The plate was incubated for 10 min at room temperature and then 50 μL of NaOH 6M were added to all wells. After exactly 10 min incubation at room temperature, the absorbance was read spectrophotometrically at 450 nm in an automatic reader (Biotek Instruments Inc., Winooski, USA). All the samples were tested in duplicate. The concentration of protein carbonyls was quantified using the molar absorption coefficient of 22,000 M^−1^ cm^−1^ [39].

### Measurement of total antioxidant capacity

Total antioxidant capacity was assessed using the antioxidant assay kit, according to the manufacturer’s instructions (Cayman Chemical, Ann Arbor, MI). This protocol assesses the combined activities of enzymatic and non-enzymatic compounds of the antioxidant system.

Amniochorion membrane fragments of 25 mg were prepared for the assay by grinding with liquid nitrogen and homogenization with 1 mL of PBS containing the anti-protease cocktail. The assay is based on the ability of the antioxidants presented in the homogenate to inhibit the oxidation of 2,2′-azino-di-[3-ethylbenzthiazoline sulfonate] (ABTS) to ABTS^+^ by metmyoglobin. The amount of ABTS^+^ produced was assessed spectrophotometrically at 405 nm. The antioxidant capacity in the sample to prevent ABTS oxidation was compared with Trolox, a water-soluble tocopherol analogue, and results were expressed as millimolar Trolox equivalent (mM Trolox). All the samples were tested in duplicate. The minimum detectable TAC level for assays was 0.004 mM.

### Statistical analysis

The Kolmogorov-Smirnov test was used to check the normality of the data. Regarding sociodemographic and obstetric variables, ethnicity, parity and Apgar 1 were compared between the groups using the Fisher exact test, while the variables maternal age, gestational age and newborn weight were compared using the nonparametric Kruskall-Wallis test.

The levels of 3-NT, carbonyl and TAC were compared among PTB, pPROM and term groups using the nonparametric Kruskall-Wallis test and between presence and absence of HC in each group using the nonparametric Mann-Whitney test. A *p* value <0.05 was considered statistically significant. The software used was SigmaStat Software version 3.1.

## Results

### Sociodemographic characteristics

Sociodemographic, obstetric and neonatal characteristics of the patients included in the study are presented in Table 1. No statistically significant differences were verified in maternal age, ethnicity, parity and Apgar 1 among the groups studied. As expected, given the study design, gestational age at delivery (*p* < 0.001) and newborn weight (*p* < 0.001) were statistically higher in the term group compared with the PTB and pPROM groups**.**

**Table 1:** Sociodemographic, obstetric and neonatal characteristics of the study population

Characteristics		PTB (*n* = 27)	pPROM (*n* = 27)	TERM (*n* = 30)	*p*-value
Maternal age (years)*		21 (19.25–24.0)	24 (17.5–32.0)	23 (20.0–27.25)	NS
Ethnicity	**White**	33.3% (9/27)	22.2% (6/27)	13.3% (4/30)	NS
	**Non-white**	66.7% (18/27)	77.8% (21/27)	86.7% (26/30)	NS
Gestational age (days)*		245 (165–256)^a^	238 (182–252)^a^	273 (259–294)^b^	<0.001
Newborn weight (g)*		2340 (630–3085)^a^	2255 (760–3550)^a^	3300 (2600–4690)^b^	<0.001
Parity	**Primiparous**	37% (10/27)	48.1% (13/27)	33.3% (10/30)	NS
Apgar 1° min	**<7**	18.5% (5/27)	14.8% (4/27)	3.33% (3/30)	NS

Values followed by the same letter do not differ

*NS* not significant

*Values expressed as median(min-max)

### Histopathological findings

Histologic chorioamnionitis was present in 22.2% (6/27) of the PTB group, 51.8% (14/27) of the pPROM group and 13.3% (4/30) of the term group. Following stratification according to histologic chorioamnionitis grading, moderate/severe cases were observed in 66% (4/6) in the PTB group, 21% (3/14) in the pPROM group and 25% (1/4) in the term group.

### Oxidative stress and antioxidant capacity

#### Quantitation of protein damage markers and total antioxidant capacity in amniochorion membranes

**Fig. 1:**
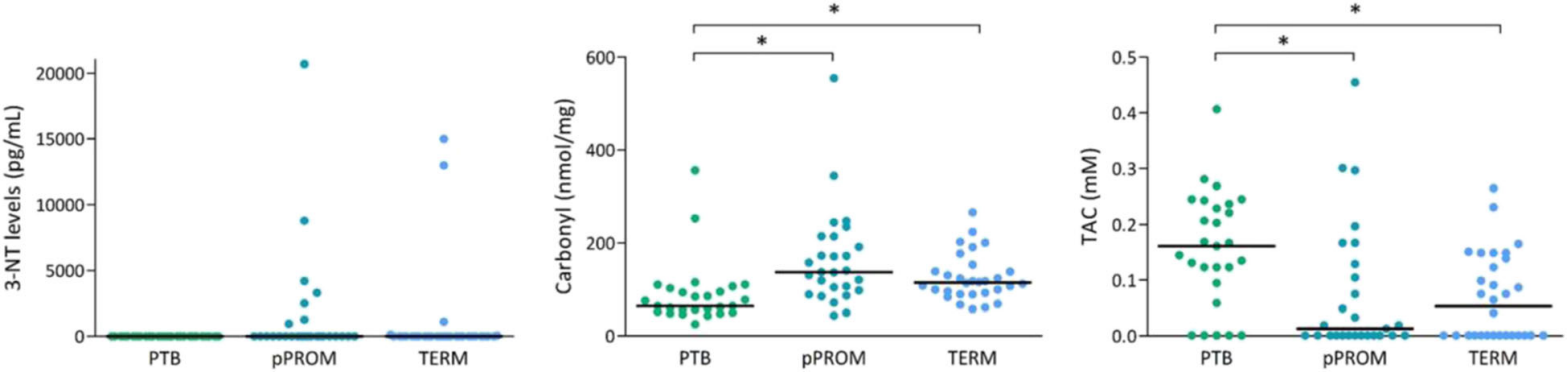
Levels of 3-NT (pg/mL), carbonyl (nmol/mg) and TAC (mM) in amniochorion membranes from the PTB, pPROM and term groups. Horizontal bars represent the median values. Kruskal-Wallis test. *p < 0.05. *Abbreviations*: 3-NT, 3-nitrotyrosine; pPROM, preterm premature rupture of membranes; PTB, preterm birth; TAC, total antioxidant capacity

3-NT concentrations, an indicator of protein peroxidation and oxidative stress, was not significantly different between the groups. However, protein oxidative damage determined by carbonyl levels was lower in the PTB group in comparison with the pPROM and term groups (*p* < 0.001). Furthermore, the PTB group had higher TAC compared with the pPROM and term groups (*p* = 0.002) (Fig. 1).

### Quantitation of protein damage markers and total antioxidant capacity in amniochorion membranes based on histologic chorioamnionitis

**Fig. 2:**
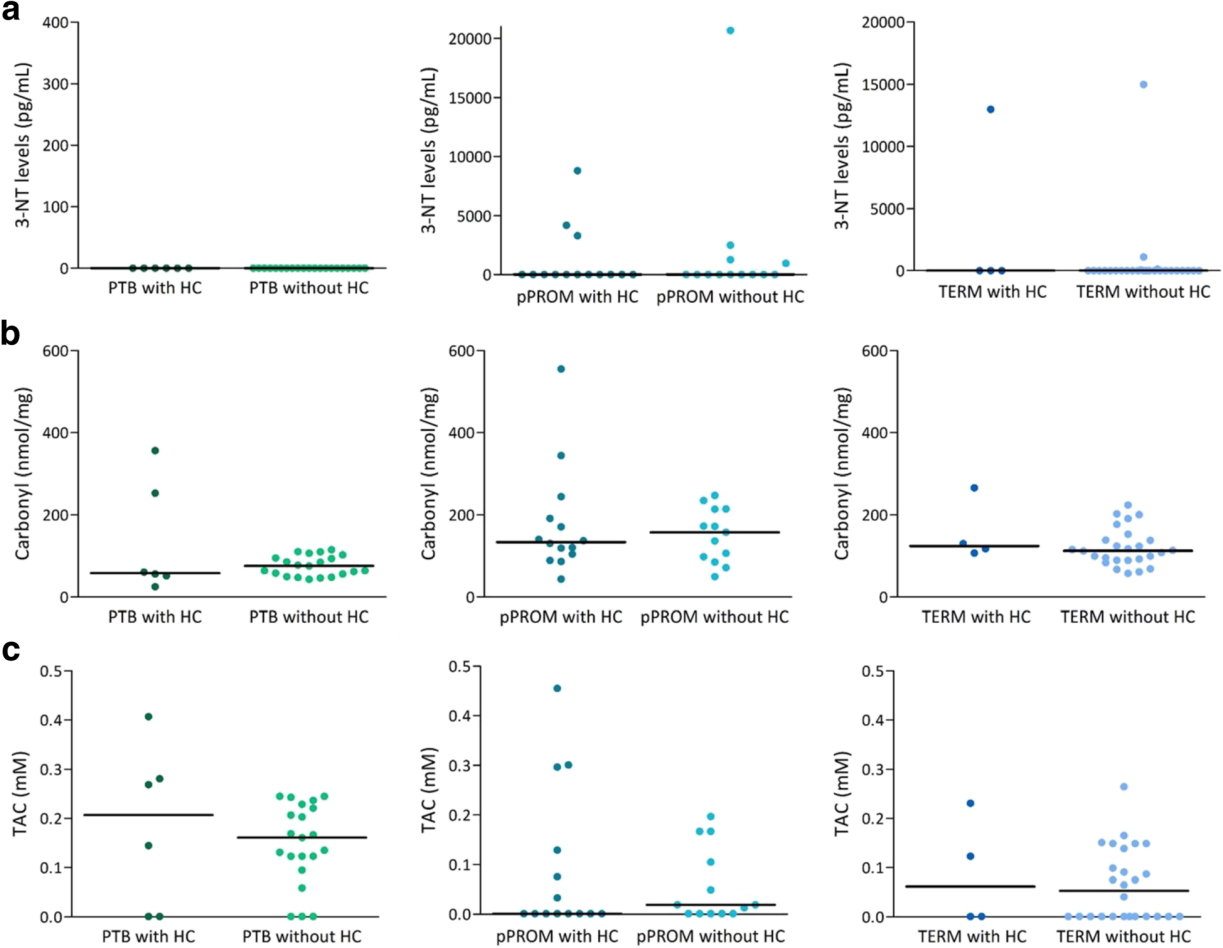
**a** Levels of 3-NT (pg/mL) in amniochorion membranes from the PTB, pPROM and term groups with and without HC. **b** Levels of carbonyl (nmol/mg) in amniochorion membranes from the PTB, pPROM and term groups with and without HC. **c** TAC (mM) in amniochorion membranes from the PTB, pPROM and term groups with and without HC. Horizontal bars represent the median values. Mann-Whitney test. *Abbreviations*: 3-NT, 3-nitrotyrosine; HC, histologic chorioamnionitis; pPROM, preterm premature rupture of membranes; PTB, preterm labor; TAC, total antioxidant capacity

Analyses of protein oxidative damage and total antioxidant capacity among the groups considering HC status are presented in Fig. 2. Protein oxidative damage assessed by 3-NT (Fig. 2a) and carbonyl (Fig. 2b) levels did not differ between the presence and absence of HC groups, regardless of gestational outcome. Similar data were obtained in relation to antioxidant capacity determined by TAC (Fig. 2c).

## Discussion

Inflammation is the physiological effecter of term parturition and the pathological initiator of labor in both PTB and pPROM. Inflammatory changes in gestational tissues result in the modification of membrane structural integrity, activation of myometrial contraction and cervical ripening that are simultaneous mechanisms responsible for the onset of labor [40]. Moreover, infection-induced inflammation and other risk factors for pPROM and PTB, including behavioral risks (e.g. cigarette smoking, alcohol and drug use), poor nutrition and obesity, can cause a redox imbalance, increasing the release of free radicals and consuming antioxidant defenses [6, 41, 42].

In this study, we demonstrated that amniochorion membranes from pregnancies complicated by pPROM showed higher protein oxidative damage and lower antioxidant capacity than those complicated by PTB. This is consistent with previous reports by Dutta et al. [25], who reported oxidative stress-induced damaged and damaged associated senescence as salient features of pPROM, but not in PTB when membranes were intact. Although there are etiological similarities between PTB and pPROM, our results also support mechanistic differences in pPROM and PTB pathways.

Menon et al. [6] reported that fetal membranes from pPROM have pronounced damage due to oxidative stress and proteolysis, whereas in PTB this oxidative damage is minimal. Compelling evidence suggests that pPROM may result from chronic oxidative stress in response to sustained exposure to risk factors, whereas PTB may be a consequence of acute oxidative stress [6, 43]. It has been suggested that an important mechanism associated with pPROM is the imbalance of redox status, where lower antioxidant status and higher oxidative stress-induced tissue damage triggers the weakening and rupture of fetal membranes. Alternately, better antioxidant capacity present in membranes from PTB can balance the acute oxidative stress allowing the cell to resist oxidative damage and membrane rupture. However, the risk factors may still cause host immune and inflammatory response sufficient to cause preterm labor. Thus, PTB pathophysiology shows a predominance of inflammatory process, while pPROM presents an intense oxidative damage of tissue, activation of senescence and senescence associated inflammation. Infection in some cases of pPROM is likely secondary, resulting from membrane damage leading to its dysfunctional status, and reduced antimicrobial resistance allowing the ascendance of microbes. Reinforcing this idea, our results showed a predominance of moderate/severe cases of HC in 66% of the PTB group, whereas mild HC was predominant in 79% of the pPROM group.

At term, oxidative stress and fetal membrane senescence are a well-described condition, probably resulting from physiological aging of the placenta and membranes that lead to labor and delivery [44, 45]. Herein, we demonstrated that the protein oxidative damage observed in pPROM amniochorion membranes was similar to that observed in term membranes. This similarity corroborates the hypothesis that elevated oxidative stress in pregnancies complicated by pPROM accelerates the premature senescence and aging, senescence-associated inflammation and proteolysis in amniochorion membranes, predisposing these to membrane rupture and the onset of labor [25, 45].

Following the evaluation of protein oxidative damage and antioxidant status in amniochorion membranes, we further determined the role of HC in the oxidative stress profile in each gestational outcome. HC was diagnosed in approximately 13% of term pregnancies and over 37% of preterm pregnancies. These data are in agreement with other studies, which demonstrated that HC is diagnosed in approximately 20% of term pregnancies and over 50% of the PTB. The majority of these cases are not associated with clinical signs and symptoms of infection [46–48].

In our study, protein oxidative damage and antioxidant capacity in amniochorion membranes did not differ based on HC status, regardless of gestational outcome. These data corroborate the findings of Kacerovsky et al. [49] and Musilova et al. [50], who observed that oxidative stress markers in pregnancies complicated by pPROM were not influenced by intra-amniotic infection, nor by HC, when measured in amniotic fluid and umbilical cord blood, respectively. These results disagree with those reported by Temma et al. [51], who showed increased oxidative stress in human placenta with HC. Similarly, a recent study by Cháfer-Pericás et al. [52] demonstrated elevated oxidative stress in amniotic fluid in the presence of intra-amniotic infection. Additionally, Perrone et al. [53] observed that HC was associated with higher oxidative stress levels in umbilical cord blood. The apparent disagreement between studies may reflect differences in the definition of the phenotype studied, the biological samples analyzed and analytes measured, as well as methodologies used in each study. It is likely that HC is a secondary phenomenon that is risk exposure and mechanism dependent, where type of host inflammatory response and biochemical (cytokines/chemokines) may determine HC outcome and thus HC is a consequence of several pathological processes.

There are limitations in our study. The first of them is the small sample size of each group when subdivided by the histopathological status of amniochorion membranes. In addition, the discrepancy between the results of the methodologies used to determine protein oxidative damage may be explained by intrinsic differences present in each technique. Determining protein carbonyl content is the most general indicator of oxidative protein damage. In addition, due to its long lasting stability of the samples under the storage conditions described, measurement of protein carbonyl content is considered a reliable marker of protein oxidation [54–56]. 3-NT quantification, in turn, is used to evaluate the protein oxidative damage more specifically, since it is a product of protein tyrosine nitration [57]. Moreover, 3-NT quantification requires sensitive analytical methods because it is typically a low-yield process [57]. Endogenous levels of 3-NT are severely low and usually close to or below the limits of detection of the currently available analytical assays [57]. Future studies by our group will examine lipid, nucleic acid and other cellular elemental damage and independent determination of multiple antioxidants.

## Conclusion

Taken together, our results suggest that increased oxidative stress and reduced antioxidant capacity are similar in amniochorion membranes from women who present pPROM and term pregnancies, reinforcing that accelerated premature senescence, senescence-associated inflammation and proteolysis predispose to pPROM. In this scenario, histologic chorioamnionitis does not modulate oxidative stress or antioxidant status profile.

## Abbreviations

3-NT: 3-nitrotyrosine
ABTS: 2,2′-azino-di-[3-ethylbenzthiazoline sulfonate]
DNPH: modified dinitrophenylhydrazine assay
ELISA: enzyme-linked immunosorbent assay
GPx: glutathione peroxidase
GSR: glutathione reductase
HC: histologic chorioamnionitis
PBS: phosphate buffered saline
PMN: polymorphonuclear cell
pPRPM: preterm premature rupture of membranes
PTB: spontaneous preterm birth
RNS: reactive nitrogen species
ROS: reactive oxygen species
SOD: superoxide dismutase
TAC: total antioxidant capacity

## References

[1] Longini M, Perrone S, Vezzosi P, Marzocchi B, Kenanidis A, Centini G (2007). Association between oxidative stress in pregnancy and preterm premature rupture of membranes. Clin Biochem.

[2] Sies H. Oxidative stress: from basic research to clinical application. Am J Med. 1991;91 10.1016/0002-9343(91)90281-2 1928209

[3] Finkel T. Signal transduction by reactive oxygen species. J Cell Biol. 2011. p. 7–15. 10.1083/jcb.201102095 PMC3135394 21746850

[4] Cadenas E, Sies H (1985). Oxidative stress: excited oxygen species and enzyme activity. Adv Enzym Regul.

[5] Ďuračková Z. Some current insights into oxidative stress. Physiol Res. 2010;59(4):459–69. 10.33549/physiolres.931844 19929132

[6] Menon R (2014). Oxidative stress damage as a detrimental factor in preterm birth pathology. Front Immunol.

[7] Chai M, Barker G, Menon R, Lappas M (2012). Increased oxidative stress in human fetal membranes overlying the cervix from term non-labouring and post labour deliveries. Placenta.

[8] Al-Gubory KH, Fowler PA, Garrel C (2010). The roles of cellular reactive oxygen species, oxidative stress and antioxidants in pregnancy outcomes. Int J Biochem Cell Biol.

[9] Agarwal A, Gupta S, Sharma RK (2005). Role of oxidative stress in female reproduction. Reprod Biol Endocrinol.

[10] Halliwell B (2012). Free radicals and antioxidants: updating a personal view. Nutr Rev.

[11] Agarwal A, Aponte-Mellado A, Premkumar BJ, Shaman A, Gupta S (2012). The effects of oxidative stress on female reproduction: a review. Reprod Biol Endocrinol.

[12] Halliwell B, Whiteman M (2004). Measuring reactive species and oxidative damage in vivo and in cell culture: how should you do it and what do the results mean?. Br J Pharmacol.

[13] Sies H (1993). Strategies of antioxidant defense. Eur J Biochem.

[14] de Vos LC, Lefrandt JD, Dullaart RPF, Zeebregts CJ, Smit AJ (2016). Advanced glycation end products: an emerging biomarker for adverse outcome in patients with peripheral artery disease. Atherosclerosis.

[15] Yeh J-K, Wang C-Y. Telomeres and telomerase in cardiovascular diseases. Genes (Basel). 2016;7:58. Available from: http://www.mdpi.com/2073-4425/7/9/58. 10.3390/genes7090058 PMC5042389 27598203

[16] Katakwar P, Metgud R, Naik S, Mittal R (2016). Oxidative stress marker in oral cancer: a review. J Cancer Res Ther.

[17] Zhou L, Wen J, Huang Z, Nice EC, Huang C, Zhang H, et al. Redox proteomics screening cellular factors associated with oxidative stress in hepatocarcinogenesis. Prot Clin Appl. 2017;(11):1–49. 10.1002/prca.201600089 27763721

[18] Udensi UK TP (2009). Oxidative stress in prostate hypertrophy and carcinogenesis. Postepy Hig Med Dosw (Online).

[19] Hussain T, Tan B, Yin Y, Blachier F, Tossou MCB, Rahu N. Oxidative Stress and Inflammation: What Polyphenols Can Do for Us? Oxid Med Cell Longev. 2016;2016. doi: 10.1155/2016/7432797. 10.1155/2016/7432797 PMC5055983 27738491

[20] Radi E, Formichi P, Battisti C, Federico A. Apoptosis and oxidative stress in neurodegenerative diseases; in : Journal of Alzheimer’s Disease. 2014, pp S125–S152.ᅟ 10.3233/JAD-132738 25056458

[21] Moreto F, Kano HT, Torezan GA, de Oliveira EP, Manda RM, Teixeira O, et al. Changes in malondialdehyde and C-reactive protein concentrations after lifestyle modification are related to different metabolic syndrome-associated pathophysiological processes. Diabetes Metab Syndr 2015;9:218–222. 10.1016/j.dsx.2015.04.008 25956753

[22] Bjørklund G, Chirumbolo S (2017). Role of oxidative stress and antioxidants in daily nutrition and human health. Nutrition.

[23] Poston L, Igosheva N, Mistry HD, Seed PT, Shennan AH, Rana S (2011). Role of oxidative stress and antioxidant supplementation in pregnancy. Am J Clin Nutr.

[24] Poston L, Raijmakers MTM (2004). Trophoblast oxidative stress, antioxidants and pregnancy outcome - a review. Placenta.

[25] Dutta EH, Behnia F, Boldogh I, Saade GR, Taylor BD, Kacerovský M (2015). Oxidative stress damage-associated molecular signaling pathways differentiate spontaneous preterm birth and preterm premature rupture of the membranes. Mol Hum Reprod.

[26] Murtha AP, Menon R (2015). Regulation of fetal membrane inflammation: a critical step in reducing adverse pregnancy outcome. Am J Obstet Gynecol.

[27] DiGiulio DB, Romero R, Amogan HP, Kusanovic JP, Bik EM, Gotsch F (2008). Microbial prevalence, diversity and abundance in amniotic fluid during preterm labor: a molecular and culture-based investigation. PLoS One.

[28] Nguyen DP, Gerber S, Hohlfeld P, Sandrine G, Witkin SS (2004). Mycoplasma hominis in mid-trimester amniotic fluid: relation to pregnancy outcome. J Perinat Med.

[29] Perni SC, Vardhana S, Korneeva I, Tuttle SL, Paraskevas LR, Chasen ST (2004). Mycoplasma hominis and Ureaplasma urealyticum in midtrimester amniotic fluid: association with amniotic fluid cytokine levels and pregnancy outcome. Am J Obstet Gynecol.

[30] Yoon B, Romero R, Lim J-H, Shim S-S, Hong J-S, Shim J-Y (2003). The clinical significance of detecting Ureaplasma urealyticum by the polymerase chain reaction in the amniotic fluid of patients with preterm labor. Am J Obstet Gynecol.

[31] Romero R, Quintero R, Oyarzun E (1988). Intraamniotic infection and the onset of labor in preterm premature rupture of the membranes. Am J Obstet Gynecol.

[32] Redline RW, Faye-Petersen O, Heller D, Qureshi F, Savell V, Vogler C (2003). Amniotic infection syndrome: Nosology and reproducibility of placental reaction patterns. Pediatr Dev Pathol.

[33] Biswas SK. Does the interdependence between oxidative stress and inflammation explain the antioxidant paradox? Oxidative Med Cell Longev. 2016; 10.1155/2016/5698931 PMC4736408 26881031

[34] Anderson MT, Staal FJ, Gitler C, Herzenberg LA (1994). Separation of oxidant-initiated and redox-regulated steps in the NF-kappa B signal transduction pathway. Proc Natl Acad Sci U S A.

[35] Flohé L, Brigelius-Flohé R, Saliou C, Traber MG, Packer L (1997). Redox regulation of NF-kappa B activation. Free Radic Biol Med.

[36] Bittar RE, Zugaib M (2009). Indicadores de risco para o parto prematuro. Rev Bras Ginecol Obs.

[37] Caughey AB, Robinson JN, Norwitz ER (2008). Contemporary diagnosis and management of preterm premature rupture of membranes. Rev Obstet Gynecol.

[38] Colombo G, Clerici M, Garavaglia ME, Giustarini D, Rossi R, Milzani A (2016). A step-by-step protocol for assaying protein carbonylation in biological samples. J Chromatogr B Anal Technol Biomed Life Sci.

[39] Mesquita CS, Oliveira R, Bento F, Geraldo D, Rodrigues JV, Marcos JC (2014). Simplified 2,4-dinitrophenylhydrazine spectrophotometric assay for quantification of carbonyls in oxidized proteins. Anal Biochem.

[40] Romero R, Espinoza J, Gonçalves LF, Kusanovic JP, Friel LA, Nien JK (2006). Inflammation in preterm and term labour and delivery. Semin Fetal Neonatal Med.

[41] Coughlan MT, Permezel M, Georgiou HM, Rice GE (2004). Repression of oxidant-induced nuclear factor-??B activity mediates placental cytokine responses in gestational diabetes. J Clin Endocrinol Metab.

[42] Dietrich M, Block G, Norkus EP, Hudes M, Traber MG, Cross CE, et al. Smoking and exposure to environmental tobacco smoke decrease some plasma antioxidants and increase gamma-tocopherol in vivo after adjustment for dietary antioxidant intakes. Am J Clin Nutr. 2003;77(1):160–166. 10.1093/ajcn/77.1.160 12499336

[43] Menon R, Polettini J, Syed TA, Saade GR, Boldogh I (2014). Expression of 8-oxoguanine Glycosylase in human fetal membranes. Am J Reprod Immunol.

[44] Behnia F, Taylor BD, Woodson M, Kacerovsky M, Hawkins H, Fortunato SJ (2015). Chorioamniotic membrane senescence: a signal for parturition?. Am J Obstet Gynecol.

[45] Menon R, Boldogh I, Hawkins HK, Woodson M, Polettini J, Syed TA, Fortunato SJ (2014). Histological evidence of oxidative stress and premature senescence in preterm premature rupture of the human fetal membranes recapitulated in vitro. Am J Pathol.

[46] Edwards RK (2005). Chorioamnionitis and labor. Obstet Gynecol Clin North Am.

[47] Conti N, Torricelli M, Voltolini C, Vannuccini S, Clifton VL, Bloise E (2015). Term histologic chorioamnionitis: a heterogeneous condition. Eur J Obstet Gynecol Reprod Biol.

[48] Newton ER (2005). Preterm labor, preterm premature rupture of membranes, and chorioamnionitis. Clin Perinatol.

[49] Kacerovsky M, Tothova L, Menon R, Vlkova B, Musilova I, Hornychova H (2014). Amniotic fluid markers of oxidative stress in pregnancies complicated by preterm prelabor rupture of membranes. J Matern Fetal Neonatal Med.

[50] Musilova I, Tothova L, Menon R, Vlkova B, Celec P, Hornychova H (2016). Umbilical cord blood markers of oxidative stress in pregnancies complicated by preterm prelabor rupture of membranes. J Matern Neonatal Med.

[51] Temma K, Shimoya K, Zhang Q, Kimura T, Wasada K, Kanzaki T (2004). Effects of 4-hydroxy-2-nonenal, a marker of oxidative stress, on the cyclooxygenase-2 of human placenta in chorioamnionitis. Mol Hum Reprod.

[52] Cháfer-Pericás C, Stefanovic V, Sánchez-Illana Á, Escobar J, Cernada M, Cubells E (2015). Novel biomarkers in amniotic fluid for early assessment of intraamniotic infection. Free Radic Biol Med.

[53] Perrone S, Tataranno ML, Negro S, Longini M, Toti MS, Alagna MG (2016). Placental histological examination and the relationship with oxidative stress in preterm infants. Placenta.

[54] Butterfield DA, Castegna A (2003). Proteomics for the identification of specifically oxidized proteins in brain: technology and application to the study of neurodegenerative disorders. Amino Acids.

[55] Dalle-Donne I, Rossi R, Giustarini D, Milzani A, Colombo R (2003). Protein carbonyl groups as biomarkers of oxidative stress. Clin Chim Acta.

[56] Stadtman ER, Levine RL (2003). Free radical-mediated oxidation of free amino acids and amino acid residues in proteins. Amino Acids.

[57] Teixeira D, Fernandes R, Prudêncio C, Vieira M (2016). 3-Nitrotyrosine quantification methods: current concepts and future challenges. Biochimie.

